# Boolean networks using the chi-square test for inferring large-scale gene regulatory networks

**DOI:** 10.1186/1471-2105-8-37

**Published:** 2007-02-01

**Authors:** Haseong Kim, Jae K Lee, Taesung Park

**Affiliations:** 1 Interdisciplinary Program in Bioinformatics, Seoul National University, South Korea; 2 Division of Biostatistics and Epidemiology, University of Virginia, USA; 3 Department of Statistics, Seoul National University, Seoul, South Korea

## Abstract

**Background:**

Boolean network (BN) modeling is a commonly used method for constructing gene regulatory networks from time series microarray data. However, its major drawback is that its computation time is very high or often impractical to construct large-scale gene networks. We propose a variable selection method that are not only reduces BN computation times significantly but also obtains optimal network constructions by using chi-square statistics for testing the independence in contingency tables.

**Results:**

Both the computation time and accuracy of the network structures estimated by the proposed method are compared with those of the original BN methods on simulated and real yeast cell cycle microarray gene expression data sets. Our results reveal that the proposed chi-square testing (CST)-based BN method significantly improves the computation time, while its ability to identify all the true network mechanisms was effectively the same as that of full-search BN methods. The proposed BN algorithm is approximately 70.8 and 7.6 times faster than the original BN algorithm when the error sizes of the Best-Fit Extension problem are 0 and 1, respectively. Further, the false positive error rate of the proposed CST-based BN algorithm tends to be less than that of the original BN.

**Conclusion:**

The CST-based BN method dramatically improves the computation time of the original BN algorithm. Therefore, it can efficiently infer large-scale gene regulatory network mechanisms.

## Background

The advancement of high-throughput technologies, such as DNA chips, has enabled the study of interactions and regulations among genes on a genome-wide scale. Recently, many algorithms have been introduced to determine gene regulatory networks based on such high-throughput microarray data, including linear models [1,2], Boolean networks [3-6], Bayesian networks [7,8], neural networks [9], and differential equations [1,10].

In the linear modeling of a genetic network, the expression data is fitted using a regression model, where the change in expression levels is a response for all other genes [1]. Although such standard linear modeling approaches enable the analysis of many different features of the modeled system, they are not effective in genome-wide network discovery. This is because the number of candidate parameters and models is very high and therefore it is difficult to search efficiently and reliably with tight control on many false positives.

Bayesian network algorithms have limitations with regard to the determination of an important network structure because of their complex modeling strategies (with a large number of parameters to be estimated) and a long computation time for searching all potential network structures on genome-wide expression data. These limitations of the Bayesian network may be overcome by the dynamic Bayesian network (DBN), which models the stochastic evolution of a set of random variables over time [11,12]. Although some improvements have been proposed, the accuracy of prediction of the DBN is relatively low, and its excessive computational time is still very high [13].

Recently, studies on the hierarchical scale-free network in lower organisms [14,15] have indicated the necessity of a network method for the simultaneous analysis of thousands of genes. The human B-cell network analysis using mutual information [16] is a type of hierarchical scale-free analysis. Although this analysis successfully constructs gene networks with thousands of genes, the method is based on mutual information between two genes; therefore, it cannot obtain the response of a target gene when more than two genes simultaneously affect the target gene.

Among these methods, the Boolean network (BN) is useful to construct gene regulatory networks observed by high-throughput microarray data because it can monitor the dynamic behavior in complex systems based on its binarization of such massive expression profiling data [17,18]. The Boolean function of a gene in a BN can describe the behavior of the target gene according to simultaneous changes in the expression levels of the other genes.

### Boolean networks

BN models were first introduced by Kauffman [3]. In these models, a gene expression is simplified with two levels: ON and OFF. A BN *G*(*V*, *F*) is defined by a set of node *V *= {*x*_1_, ..., *x*_*n*_} and a set of Boolean functions *F *= {*f*_1_, ..., *f*_*n*_}. A Boolean function *f*_*i*_(*x*_1_, ..., *x*_*k*_), where *i *= {1, ..., *n*}, with *k *specified input nodes (indegree) is assigned to node *x*_*i*_. The regulation of nodes is defined by *F*. More specifically, given the values of the node (*V*) at time *t *- 1, the Boolean function are used to update these values at time *t*.

The model system has been developed into a so-called Random BN model [19]. BNs have attracted attention since the introduction of probabilistic Boolean network (PBN) models by Shmulevich *et al*. [6]. Many algorithms have been proposed for the inference of BNs. For example, the REVEAL algorithm has been introduced by Liang *et al*. [5] for causal inference by using mutual information, which is the most fundamental and general measure of correlation. Akutsu *et al*. [4] have constructed a BN structure based on the consistency problem, which can be used to determine the existence of a network that is consistent with the observed data. In one of the most recent studies on the BN algorithm, the Best-Fit Extension problem [20] is used for the inference of PBNs [6]. In PBNs, every node (gene) can have a chance of acquiring different Boolean functions. The probabilistic selection of Boolean functions adds flexibility in the determination of the steady state of BNs and monitoring of the dynamical network behavior for gene perturbation or intervention [18,21].

Recently, several software packages have been developed for constructing BNs. The random BN toolbox [22] and the PBN toolbox [6] are available in Matlab. NetBuilder (version 0.94) [23] is a genetic regulatory network tool used to simulate genetic network using a BN. The BN has been widely used to describe biological processes. For example, Huang [17] has used these networks to represent cell growth, cell differentiation, and apoptosis. A transcriptional network model in yeast has been studied using a random BN [24]. Further, Johnson [25] has studied the signal transduction pathways in a B-cell ligand screen.

### Advantages and disadvantages of Boolean networks

The estimation of gene regulatory networks using the BN offers several advantages. First, the BN model effectively explains the dynamic behavior of living systems [17,18,26]. Simplistic Boolean formalism can represent realistic complex biological phenomena such as cellular state dynamics that exhibit switch-like behavior, stability, and hysteresis [17]. It also enables the modeling of non-linear relations in complex living systems [27]. Second, Boolean algebra is an established science that provides a large set of algorithms that are already available for supervised learning in the binary domain, such as the logical analysis of data [28], and Boolean-based classification algorithms [29]. Finally, dichotomization to binary values improves the accuracy of classification and simplifies the obtained models by reducing the noise level in experimental data [30,31].

However, the BN has some drawbacks. One of the major drawbacks is that it requires extremely high computing times to construct reliable network structures. Therefore, most BN algorithms such as REVEAL can thus be used only with a small number of genes and a low indegree value. For higher indegree values, these algorithms should be accelerated through parallelization in order to increase the search efficiency in the solution space [5]. The consistency problem [4] works in time complexity *O*(22k
 MathType@MTEF@5@5@+=feaafiart1ev1aaatCvAUfKttLearuWrP9MDH5MBPbIqV92AaeXatLxBI9gBaebbnrfifHhDYfgasaacH8akY=wiFfYdH8Gipec8Eeeu0xXdbba9frFj0=OqFfea0dXdd9vqai=hGuQ8kuc9pgc9s8qqaq=dirpe0xb9q8qiLsFr0=vr0=vr0dc8meaabaqaciaacaGaaeqabaqabeGadaaakeaacqaIYaGmdaahaaWcbeqaaiabikdaYmaaCaaameqabaGaem4AaSgaaaaaaaa@304A@·(nk)
 MathType@MTEF@5@5@+=feaafiart1ev1aaatCvAUfKttLearuWrP9MDH5MBPbIqV92AaeXatLxBI9gBaebbnrfifHhDYfgasaacH8akY=wiFfYdH8Gipec8Eeeu0xXdbba9frFj0=OqFfea0dXdd9vqai=hGuQ8kuc9pgc9s8qqaq=dirpe0xb9q8qiLsFr0=vr0=vr0dc8meaabaqaciaacaGaaeqabaqabeGadaaakeaadaqadaqaauaabeqaceaaaeaacqWGUbGBaeaacqWGRbWAaaaacaGLOaGaayzkaaaaaa@3106@·*m*·*n*·*poly*(*k*)) (*m *is the number of observed time points; *n*, the total number of genes; and *poly*(*k*), the time required to compare a pair of examples respectively) for a fixed indegree *k*; this is because 22k
 MathType@MTEF@5@5@+=feaafiart1ev1aaatCvAUfKttLearuWrP9MDH5MBPbIqV92AaeXatLxBI9gBaebbnrfifHhDYfgasaacH8akY=wiFfYdH8Gipec8Eeeu0xXdbba9frFj0=OqFfea0dXdd9vqai=hGuQ8kuc9pgc9s8qqaq=dirpe0xb9q8qiLsFr0=vr0=vr0dc8meaabaqaciaacaGaaeqabaqabeGadaaakeaacqaIYaGmdaahaaWcbeqaaiabikdaYmaaCaaameqabaGaem4AaSgaaaaaaaa@304A@ Boolean functions must be checked for each of the possible _*n*_*C*_*k *_combinations of variables and for *m *observations. The Best-Fit Extension problem [21] also works in time complexity *O*(22k
 MathType@MTEF@5@5@+=feaafiart1ev1aaatCvAUfKttLearuWrP9MDH5MBPbIqV92AaeXatLxBI9gBaebbnrfifHhDYfgasaacH8akY=wiFfYdH8Gipec8Eeeu0xXdbba9frFj0=OqFfea0dXdd9vqai=hGuQ8kuc9pgc9s8qqaq=dirpe0xb9q8qiLsFr0=vr0=vr0dc8meaabaqaciaacaGaaeqabaqabeGadaaakeaacqaIYaGmdaahaaWcbeqaaiabikdaYmaaCaaameqabaGaem4AaSgaaaaaaaa@304A@·(nk)
 MathType@MTEF@5@5@+=feaafiart1ev1aaatCvAUfKttLearuWrP9MDH5MBPbIqV92AaeXatLxBI9gBaebbnrfifHhDYfgasaacH8akY=wiFfYdH8Gipec8Eeeu0xXdbba9frFj0=OqFfea0dXdd9vqai=hGuQ8kuc9pgc9s8qqaq=dirpe0xb9q8qiLsFr0=vr0=vr0dc8meaabaqaciaacaGaaeqabaqabeGadaaakeaadaqadaqaauaabeqaceaaaeaacqWGUbGBaeaacqWGRbWAaaaacaGLOaGaayzkaaaaaa@3106@·*m*·*n*·*poly*(*k*)). Although the improved consistency algorithm and Best-Fit Extension problem work in time complexity *O*((nk)
 MathType@MTEF@5@5@+=feaafiart1ev1aaatCvAUfKttLearuWrP9MDH5MBPbIqV92AaeXatLxBI9gBaebbnrfifHhDYfgasaacH8akY=wiFfYdH8Gipec8Eeeu0xXdbba9frFj0=OqFfea0dXdd9vqai=hGuQ8kuc9pgc9s8qqaq=dirpe0xb9q8qiLsFr0=vr0=vr0dc8meaabaqaciaacaGaaeqabaqabeGadaaakeaadaqadaqaauaabeqaceaaaeaacqWGUbGBaeaacqWGRbWAaaaacaGLOaGaayzkaaaaaa@3106@·*m*·*n*·*poly*(*k*)) [32], they still exhibit an exponential increase in the computing time for the parameters *n *and *k*. Such high computing times are a major problem in the study of large-scale gene regulatory and gene interaction systems using BNs.

### Chi-square-test-based Boolean network

In order to overcome the time complexity problem of the BN method, we propose a variable selection method based on the chi-square test (CST). The proposed CST-based BN adopts the Best-Fit Extension problem, which is commonly used in the PBN to effectively determine all possible relevant Boolean functions. In our method, the maximum indegree of networks is assumed to be three. We also focus on the Boolean functions that comprise three different literals (input genes in the Boolean function). Each literal is connected by the three Boolean operators NOT(¬), AND(∧), OR(∨); for example, *f *= *X*1 ∧ ¬*X*2 ∨ *X*3. Then, the time complexity of the CST-based BN reduces to *O*(22k⋅∑j=1n∑i=1n1,j⋅(n2,ijk)
 MathType@MTEF@5@5@+=feaafiart1ev1aaatCvAUfKttLearuWrP9MDH5MBPbIqV92AaeXatLxBI9gBaebbnrfifHhDYfgasaacH8akY=wiFfYdH8Gipec8Eeeu0xXdbba9frFj0=OqFfea0dXdd9vqai=hGuQ8kuc9pgc9s8qqaq=dirpe0xb9q8qiLsFr0=vr0=vr0dc8meaabaqaciaacaGaaeqabaqabeGadaaakeaacqaIYaGmdaahaaWcbeqaaiabikdaYmaaCaaameqabaGaem4AaSgaaaaakiabgwSixpaaqadabaWaaabmaeaacqGHflY1daqadaqaauaabeqaceaaaeaacqWGUbGBdaWgaaWcbaGaeGOmaiJaeiilaWIaemyAaKMaemOAaOgabeaaaOqaaiabdUgaRbaaaiaawIcacaGLPaaaaSqaaiabdMgaPjabg2da9iabigdaXaqaaiabd6gaUnaaBaaameaacqaIXaqmcqGGSaalcqWGQbGAaeqaaaqdcqGHris5aaWcbaGaemOAaOMaeyypa0JaeGymaedabaGaemOBa4ganiabggHiLdaaaa@4ECE@·*m*·*poly*(*k*)), where *n *is the total number of genes, *k *is the indegree, *m *is the total number of time points, *n*_1, *j *_is the number of first selected genes for the *j*th gene, and *n*_2, *ij *_is the number of second selected genes when the *i*th gene is selected in the first step. We have found that the dichotomization of the continuous gene expression values allows us to efficiently perform the independence test for a two-way contingency table on each pairwise network mechanisms. We use the CST to identify genes that are associated with a target gene. A target gene would be expressed in accordance with a Boolean function related to the selected genes. Since the genes have only two levels, (0 and 1), we use 2 × 2 and 2 × 2 × 2 contingency tables to identify the relationship between two and three genes respectively. The proposed method is used along with the Best-Fit Extension problem. This method is described in detail in the Methods section.

## Results

### Simulation study

**Figure 1:**
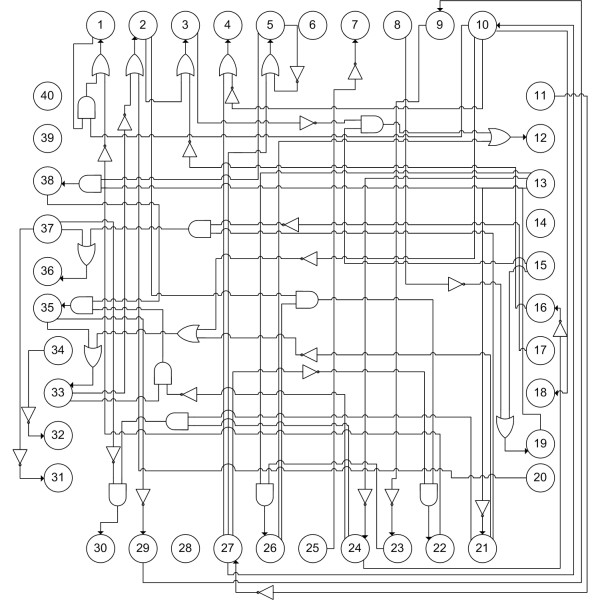
**Network structure with 40 nodes in simulation data set**. Network structure with 27 Boolean functions that are randomly generated with 40 nodes.

For our simulation study, an artificially generated network structure is illustrated in Figure 1. It comprises 40 nodes and the maximum value of the indegree (*k*) is three. The network structure is composed of 27 Boolean functions that are randomly generated from 40 nodes. Forty sets of binary data are obtained sequentially from the network structure. Each data set has different initial states and seven time points. Since the data sets are generated from definite Boolean functions, the genes in the Boolean functions tend to have strong associations. The CST-based BN uses two thresholds *α*_1 _and *α*_2 _where *α*_1 _is used for selecting variables for the main effect and *α*_2 _is used for the conditional effect. A detailed description is provided in the Methods section. The smaller the values of *α*_1 _and *α*_2_, the stronger is the association of the variables. In order to select the nodes that have strong associations with the target nodes, we used very small cutoff values – *α*_1 _= 1 - *e*15 and *α*_2 _= 1 - *e*15 – for the CST-based BN. Table 1 shows the result of the original BN and the CST-based BN for various noise levels. The random noise is added to the binary data generated sequentially by the Boolean functions. For example, if the noise level is 0.1%, a Boolean function, which generates 1 when the noise level is zero generates 1 with a probability of 99.999 and generates 0 with a probability of 0.1%. Since we have 301 time points (43 × 7) and 1 time lag, the total noise level of a gene is 2.96% (≈1 - (1 - 0.0001)^301^) when the noise level is 0.01% in a Boolean function. For the correction of the multiple comparison problem, we compare the results of the original BN and CST-based BN when the noise level of a Boolean function varies from 0.01% to 0.24%.

**Table 1:** The simulation results of the original BN and CST-based BN.

Noise level	Original BN Number of Boolean functions	FPR	CST-based BN Number of Boolean functions	FPR
0	348(27)*	0.922	286(27)	0.899
0.01%	326(22)	0.932	264(22)	0.916
0.02%	269(22)	0.918	207(22)	0.893
0.04%	313(20)	0.936	289(20)	0.930
0.06%	264(18)	0.931	244(18)	0.926
0.08%	168(12)	0.928	139(12)	0.913
0.1%	271(17)	0.937	216(15)	0.930
0.12%	243(16)	0.934	209(16)	0.923
0.14%	614(19)	0.969	514(19)	0.963
0.16%	73(9)	0.876	73(9)	0.876
0.18%	269(15)	0.944	217(15)	0.930
0.2%	198(9)	0.954	167(9)	0.946
0.22%	54(5)	0.907	54(5)	0.907
0.24%	369(3)	0.991	318(3)	0.990

* Total number of Boolean functions (Number of true Boolean functions).

**Figure 2:**
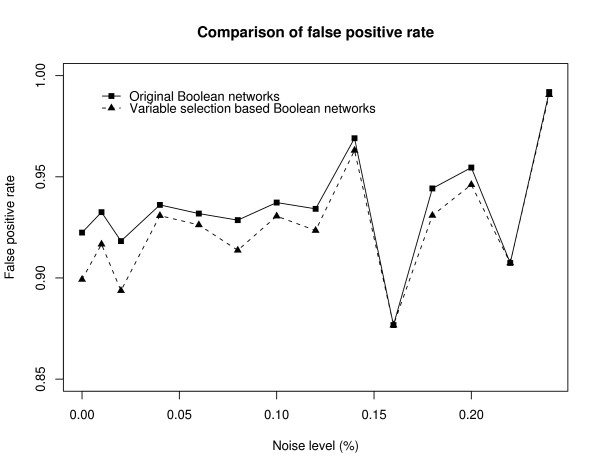
**False positive rates**. The result obtained by the CST-based BN is more accurate than that obtained by the original BN. It appears that the Boolean networks determine Boolean functions with significantly higher false positive genes than those in the case of the proposed variable selection method.

Table 1 summarizes the simulation result. The first column shows the noise level and the second column shows the number of Boolean functions identified by the original BN as well as the number of true Boolean functions in parentheses. The third column shows the result of the CST-based BN. The number of the Boolean functions provided by CST-based BN is the same as that provided by the original BN. The false positive rate (FPR) is defined as the ratio of the number of false Boolean functions to the total number of Boolean functions. For example, when the noise level is 0, the FPR of the original BN is given by FPR = (348 - 27)/348 = 0.922. Figure 2 shows the FPRs for various noise levels. It appears that the CST-based BN reduces the FPR of the original BN.

The CST-based BN method yielded the same estimated Boolean functions as those obtained by the original BN method for various noise levels. However, there are large differences between the computing times. Table 2 shows the selected number of nodes in the first and second steps of the variable selection method. The first column shows the node number. The second and third columns show the number of nodes in the first and second steps, respectively. Each number in the third column, separated by a comma, is the number of selected nodes for each node selected in the first step. For example, in the second line of Table 2, there are two selected nodes in the first step. For these two nodes, there are 39 and 38 selected nodes in the second step, respectively. From the result of the variable selection (Table 2), the ratio of the time complexities of the two methods can be obtained as follows:

**Table 2:** The selected number of nodes in the first and second steps of variable selection

Node number	Number of selected nodes at the first step	Number of selected nodes at the second step
1	1	39
2	2	39, 38
3	1	39
4	1	39
5	1	39
6	0	0
7	8	39, 37, 36, 36, 32, 32, 33, 32
8	1	39
9	4	39, 37, 37, 35
10	3	39, 38, 37
11	0	0
12	9	39, 38, 37, 36, 32, 32, 33, 32, 31
13	0	0
14	0	0
15	0	0
16	4	39, 37, 37, 36
17	0	0
18	3	39, 38, 37
19	1	39
20	0	0
21	5	39, 37, 37, 36, 35
22	5	39, 37, 37, 36, 35
23	2	39, 38
24	5	39, 37, 37, 36, 35
25	0	0
26	0	0
27	5	39, 38, 37, 33, 33
28	0	0
29	1	39
30	7	39, 34, 35, 35, 35, 34, 33
31	10	39, 34, 33, 32, 31, 32, 33, 32, 31, 30
32	8	39, 34, 33, 32, 33, 33, 33, 32
33	1	39
34	0	0
35	1	39
36	2	39, 38
37	0	0
38	1	39
39	0	0
40	0	0

Time complexity of original BNTime complexity of CST-based BN=O(22k⋅(nk)⋅n⋅m⋅poly(k))O(22k⋅∑j=1n∑i=1n1,j⋅(n2,ijk)⋅m⋅poly(k))=(nk)⋅n∑j=1n∑i=1n1,j⋅(n2,ijk)=6.8865
 MathType@MTEF@5@5@+=feaafiart1ev1aaatCvAUfKttLearuWrP9MDH5MBPbIqV92AaeXatLxBI9gBaebbnrfifHhDYfgasaacH8akY=wiFfYdH8Gipec8Eeeu0xXdbba9frFj0=OqFfea0dXdd9vqai=hGuQ8kuc9pgc9s8qqaq=dirpe0xb9q8qiLsFr0=vr0=vr0dc8meaabaqaciaacaGaaeqabaqabeGadaaakeaafaqaaeWabaaabaWaaSaaaeaacqqGubavcqqGPbqAcqqGTbqBcqqGLbqzcqqGGaaicqqGJbWycqqGVbWBcqqGTbqBcqqGWbaCcqqGSbaBcqqGLbqzcqqG4baEcqqGPbqAcqqG0baDcqqG5bqEcqqGGaaicqqGVbWBcqqGMbGzcqqGGaaicqqGVbWBcqqGYbGCcqqGPbqAcqqGNbWzcqqGPbqAcqqGUbGBcqqGHbqycqqGSbaBcqqGGaaicqqGcbGqcqqGobGtaeaacqqGubavcqqGPbqAcqqGTbqBcqqGLbqzcqqGGaaicqqGJbWycqqGVbWBcqqGTbqBcqqGWbaCcqqGSbaBcqqGLbqzcqqG4baEcqqGPbqAcqqG0baDcqqG5bqEcqqGGaaicqqGVbWBcqqGMbGzcqqGGaaicqqGdbWqcqqGtbWucqqGubavcqqGTaqlcqqGIbGycqqGHbqycqqGZbWCcqqGLbqzcqqGKbazcqqGGaaicqqGcbGqcqqGobGtaaaabaGaeyypa0ZaaSaaaeaacqWGpbWtcqGGOaakcqaIYaGmdaahaaWcbeqaaiabikdaYmaaCaaameqabaGaem4AaSgaaaaakiabgwSixpaabmaabaqbaeqabiqaaaqaaiabd6gaUbqaaiabdUgaRbaaaiaawIcacaGLPaaacqGHflY1cqWGUbGBcqGHflY1cqWGTbqBcqGHflY1cqWGWbaCcqWGVbWBcqWGSbaBcqWG5bqEcqGGOaakcqWGRbWAcqGGPaqkcqGGPaqkaeaacqWGpbWtcqGGOaakcqaIYaGmdaahaaWcbeqaaiabikdaYmaaCaaameqabaGaem4AaSgaaaaakiabgwSixpaaqadabaWaaabmaeaacqGHflY1daqadaqaauaabeqaceaaaeaacqWGUbGBdaWgaaWcbaGaeGOmaiJaeiilaWIaemyAaKMaemOAaOgabeaaaOqaaiabdUgaRbaaaiaawIcacaGLPaaaaSqaaiabdMgaPjabg2da9iabigdaXaqaaiabd6gaUnaaBaaameaacqaIXaqmcqGGSaalcqWGQbGAaeqaaaqdcqGHris5aaWcbaGaemOAaOMaeyypa0JaeGymaedabaGaemOBa4ganiabggHiLdGccqGHflY1cqWGTbqBcqGHflY1cqWGWbaCcqWGVbWBcqWGSbaBcqWG5bqEcqGGOaakcqWGRbWAcqGGPaqkcqGGPaqkaaaabaGaeyypa0ZaaSaaaeaadaqadaqaauaabeqaceaaaeaacqWGUbGBaeaacqWGRbWAaaaacaGLOaGaayzkaaGaeyyXICTaemOBa4gabaWaaabmaeaadaaeWaqaaiabgwSixpaabmaabaqbaeqabiqaaaqaaiabd6gaUnaaBaaaleaacqaIYaGmcqGGSaalcqWGPbqAcqWGQbGAaeqaaaGcbaGaem4AaSgaaaGaayjkaiaawMcaaaWcbaGaemyAaKMaeyypa0JaeGymaedabaGaemOBa42aaSbaaWqaaiabigdaXiabcYcaSiabdQgaQbqabaaaniabggHiLdaaleaacqWGQbGAcqGH9aqpcqaIXaqmaeaacqWGUbGBa0GaeyyeIuoaaaGccqGH9aqpcqaI2aGncqGGUaGlcqaI4aaocqaI4aaocqaI2aGncqaI1aqnaaaaaa@F955@

In summary, the CST-based BN method was approximately 6.9 times faster than the original BN method. If the network had a larger number of nodes (*n*), then the difference between the computing times of the two algorithms would be significantly high.

### Yeast cell cycle data

In order to demonstrate the improvement in the computing times, we apply the proposed variable selection method to yeast cell cycle data [33]. The data comprises 18 time points (alpha-factor-based synchronization experiment). In this example, the computing time and accuracy of network estimation of the original BN method are compared with those of our CST-based BN method.

### Comparison between the network structure estimation accuracies of the CST-based BN and the original BN

Our variable selection method significantly improves the computing time of the BNs. However, the accuracy of our method should be assessed before comparing the computing times of the two methods. The improvement in the computing times primarily depends on the cutoff statistical significance levels, *α*_1 _and *α*_2_, for the selection of genes at time *t *- 1 via the CST (Methods section). Depending on the choice of appropriate values of *α*_1 _and *α*_2_, the proposed CST-based BN method may not be able to determine some Boolean functions that can be estimated by the original BN method. This may be attributed to the missing essential variables due to the usage of extremely stringent cutoff values.

**Figure 3:**
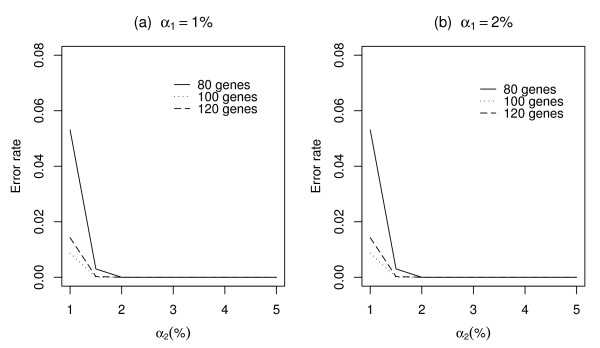
**Error rates of estimated Boolean functions using variable selection method when the error size is 0**. The error rates were calculated from the three data sets with a different number of genes: 80, 100, and 120. The genes were randomly selected from the yeast cell cycle data set. (a) and (b) show the plots when the cutoff values are *α*_1 _= 1% and *α*_1 _= 2%, respectively. The appropriate values of *α*_1 _and *α*_2 _are 1% and 2% when the error size is 0.

**Figure 4:**
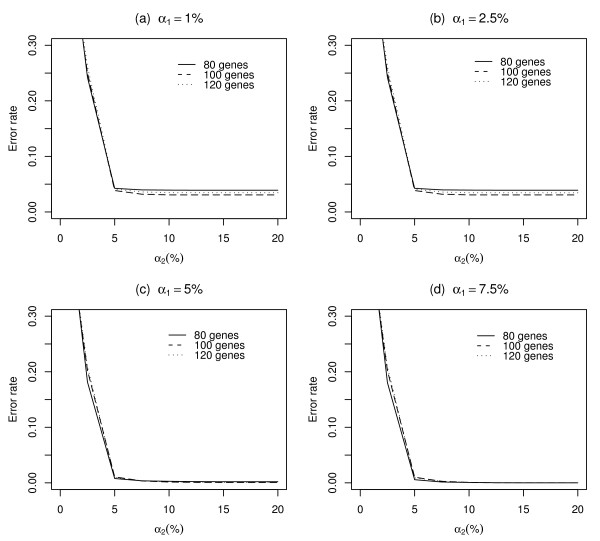
**Error rates of estimated Boolean functions using variable selection method when the error size is 1**. The error rates were calculated from the three data sets with a different number of genes: 80, 100, and 120. The genes were randomly selected from the yeast cell cycle data set. (a), (b), (c), and (d) show the plots when the cutoff values of *α*_1 _are 1%, 2.5%, 5%, and 7.5%, respectively. The appropriate values of *α*_1 _and *α*_2 _are 7.5% and 10%, respectively, when the error size is 1.

We define the error rate as the discrepancy between the Boolean functions estimated by the original BN and the proposed CST-based BN as follows:

Error rate=1−Number of (BFOriginalBN∩BFCSTbasedBN)Number of (BFOriginalBN)     (1)
 MathType@MTEF@5@5@+=feaafiart1ev1aaatCvAUfKttLearuWrP9MDH5MBPbIqV92AaeXatLxBI9gBaebbnrfifHhDYfgasaacH8akY=wiFfYdH8Gipec8Eeeu0xXdbba9frFj0=OqFfea0dXdd9vqai=hGuQ8kuc9pgc9s8qqaq=dirpe0xb9q8qiLsFr0=vr0=vr0dc8meaabaqaciaacaGaaeqabaqabeGadaaakeaacqqGfbqrcqqGYbGCcqqGYbGCcqqGVbWBcqqGYbGCcqqGGaaicqqGYbGCcqqGHbqycqqG0baDcqqGLbqzcqGH9aqpcqaIXaqmcqGHsisldaWcaaqaaiabb6eaojabbwha1jabb2gaTjabbkgaIjabbwgaLjabbkhaYjabbccaGiabb+gaVjabbAgaMjabbccaGiabcIcaOiabdkeacjabdAeagnaaBaaaleaacqWGpbWtcqWGYbGCcqWGPbqAcqWGNbWzcqWGPbqAcqWGUbGBcqWGHbqycqWGSbaBcqWGcbGqcqWGobGtaeqaaOGaeyykICSaemOqaiKaemOray0aaSbaaSqaaiabdoeadjabdofatjabdsfaujabdkgaIjabdggaHjabdohaZjabdwgaLjabdsgaKjabdkeacjabd6eaobqabaGccqGGPaqkaeaacqqGobGtcqqG1bqDcqqGTbqBcqqGIbGycqqGLbqzcqqGYbGCcqqGGaaicqqGVbWBcqqGMbGzcqqGGaaicqGGOaakcqWGcbGqcqWGgbGrdaWgaaWcbaGaem4ta8KaemOCaiNaemyAaKMaem4zaCMaemyAaKMaemOBa4MaemyyaeMaemiBaWMaemOqaiKaemOta4eabeaakiabcMcaPaaacaWLjaGaaCzcamaabmaabaGaeGymaedacaGLOaGaayzkaaaaaa@8AF0@

where *BF*_*OriginalBN *_and *BF*_*CSTbasedBN *_are sets of Boolean functions estimated by the original BN and the CST-based BN, respectively. Three data sets with randomly selected 80, 100, and 120 genes were used to compute the error rate. We have calculated the error rate for various values of *α*_1 _and *α*_2 _(Figure 3(a),(b) and Figure 4(a)–(d)). Figure 3 shows the error rate when the error size of the Best-Fit Extension problem is 0, while Figure 4 shows the error rate when the error size is 1. In each plot, the *y*-axis represents the error rate and the *x*-axis represents the value of *α*_2_. The error rate decreases with an increase in *α*_1 _and *α*_2_; this implies that a less conservative cutoff value would be more suitable to construct a BN.

In order to select appropriate values of *α*_1 _and *α*_2_, the error rates were obtained for various combination of (*α*_1_, *α*_2_). Some of the results are shown in Figures 3 and 4. To obtain an error rate of zero, the values of *α*_1 _and *α*_2 _should be greater than 1% and 2%, respectively, when the error size is zero (Figure 3(a),(b)). However, when the error size is one, larger values of *α*_1 _and *α*_2 _are required to obtain an error rate of zero (Figure 4(a)–(d)). Based on these results, we suggest that the following values be used when the error size is 0, *α*_1 _= 1%, *α*_2 _= 2% and when the error size is 1, *α*_1 _= 7.5%, *α*_2 _= 10%.

### Comparison between the computing times of the original and the proposed BN algorithms

**Figure 5:**
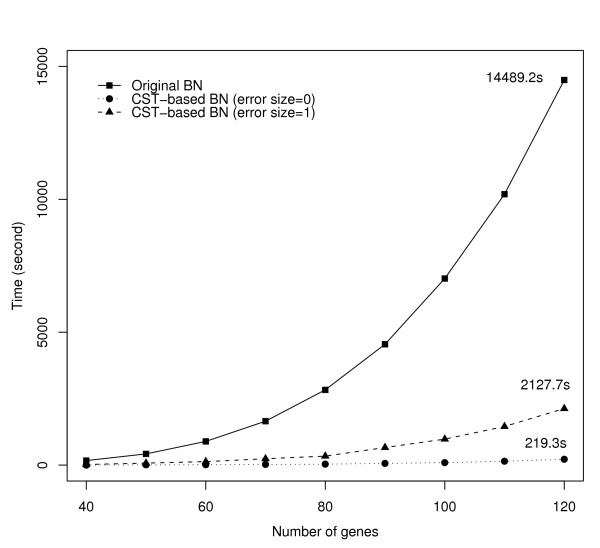
**Change in the computation times when the total number of genes varies from 40 to 120 for *k *= 3**. The line with circles represents the computation times of the original BN. The dotted line with rectangles and the dashed line with triangles indicate the computation times of the CST-based BN when the error sizes are 0 and 1, respectively. The computation times of the proposed method are approximately 70.8 and 7.6 times faster than those of the original BN method when the error sizes are 0 and 1, respectively.

In order to compare the computing times, we executed the BN program based on the Best-Fit Extension problem (written in C language). Figure 5 shows the computing times with a change in the number of genes from 40 to 120. We set the value of error size in the Best-Fit Extension problem as 0 and 1 and used the variable selection criteria *α*_1 _= 1%, *α*_2 _= 2% and *α*_1 _= 7.5%, *α*_2 _= 10%, respectively. The line with circles represents the computation time of the original BN. The dotted line with rectangles and the dashed line with triangles represent the computation times of the CST-based BN when the error size are 0 and 1, respectively. As shown in Figure 5, the original BN required 14489.2 s to estimate all Boolean functions with 120 genes for *k *= 3. On the other hand, the proposed CST-based BN required only 219.3 s for *α*_1 _= 1%, *α*_2 _= 2% and 2127.7 s for *α*_1 _= 7.5%, *α*_2 _= 10%. Therefore, the overall computation times of the proposed CST-based BN are approximately 70.8 times (error size = 0) and 7.6 times (error size = 1) faster than those of the original BN method.

### Construction of gene networks with yeast cell cycle related 800 genes

**Figure 6:**
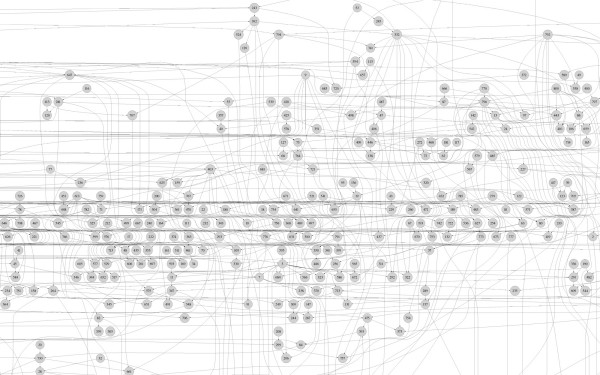
**Gene network structure with 800 genes**. 800 gene network structure with yeast cell cycle using CST-based BN. The cutoff values are *α*_1 _= 1% and *α*_2 _= 2%. This structure is an example of many possible structures. Only a partial structure is presented.

We also applied the CST-based BN to the subset of the yeast cell cycle data with 800 genes [33] for demonstration. Figure 6 shows a partial structure of the gene network structure constructed by using the CST-based BN. The Best-Fit Extension problem provided several Boolean functions for a gene at time *t*. For the demonstration, we randomly selected a Boolean function from the estimated Boolean functions of each gene; the method used for this purposed was similar to that used by the PBN [6,34] to select a set of Boolean functions for a given gene using the coefficient of determination (COD).

For all 800 genes, the CST-based BN required approximately three days to construct the network structure. In order to estimate the total computation time of the original BN, we selected the first target gene and constructed the BN, which required approximately 37,011 s. Therefore, the total computation times for all 800 genes will be approximately 342 days to build the network structure. Hence, the computing time of the proposed CST-based BN method is approximately 114 times faster than that of the original BN method.

## Discussions and Conclusion

Recent studies [14,15] have emphasized that thousands of genes must be considered simultaneously in order to construct gene regulatory networks in an organism. The BN method is useful for constructing a gene regulatory network. If the gene expression data contain a considerable amount of noise, the binary transformation of these data can reduce the error [35]. The BN has been successfully used to model a nonlinear system [27] and the dynamic behavior of living systems [18,26]. Despite these advantages, it is difficult to apply the BN method to large-scale gene regulatory network studies due to the extremely high computation times.

In order to overcome this computational drawback, we proposed the variable selection method using the CST for the two-way and three-way contingency tables of Boolean count observations. This method reduces the computation times significantly; for example, for 120 genes, the computation time is approximately 70.8 times faster than that of the original BN method. If the total number of genes and the value of *k *increase, the improvement in the computation time is expected to be significantly greater than the original BN method. Also the proposed method can be easily implemented with the existing BN modeling algorithms such as the PBNs by efficiently selecting only the most relevant genes for determining the Boolean functions. This method is thus demonstrated to reduce the false positive rate, which is an important problem in network studies conducted on a genome-wide scale.

In our method, the value of *k *is assumed to be three. However, it is possible to use a large value of *k *greater than 3. Since our method uses the Best-Fit Extension problem, a gene can be controlled by more than one Boolean function. Therefore, it appears that *k *= 3 provides a large number of Boolean functions that can model the gene regulatory network successfully.

The proposed CST-based BN used the two-step discovery of 3-indegree Boolean functions because the prediction information of addtional genes in such high-dimensional Boolean functions is mainly observed after considering, or conditional on, primary genes' effects. This strategy, in turn, resulted in much more efficient discovery of the most predictive high-dimensional Boolean functions in our BN modeling.

The result of the proposed CST-based BN may be sensitive to the sample size *n*. When *n *is small, the contingency table may contain many cells with low and zero frequencies. To ensure that the expectation value is not equal to zero, a continuity correction is used by adding a small constant 0.1 to the observed frequency in each cell [36]. This simple correction produces a successful result in the real data set [33] that contains 17 time points. However, we should be more careful while applying the CST method to data with small time points because the result of the CST can be less reliable for the sparse data set. In this case, we suggest that the CST be replaced with Fisher's exact test that provides a more reliable result for the small sample size data [36]. The small sample size problem also makes it difficult for the original BN algorithm to produce a reliable result. We think that in the near future the advancement of high throughput techniques and the cost-down of microarrays will enable us to solve the sparse data problem by producing large data sets easily.

The improvement of the computing times using the CST-based BN will significantly increase the utility and applicability of the BN to the inference of various regulatory networks, particularly those based on current large screening biological data such as microarrays. In order to apply the proposed variable selection method, we must first select the values of *α*_1 _and *α*_2_. A small values of *α *cause the exclusion of essential Boolean functions and produces a high error rate. On the other hand, large values of *γ *cause the inclusion of most of the genes, thereby resulting in long computing times. For the yeast cell cycle data, we applied various combinations of (*α*_1_, *α*_2_). As shown in Figure 3, it appears that the cutoff values do not significantly affect much the accuracy of the method for the yeast cell cycle data, provided the values of *α*_1 _and *α*_2 _are greater than 1% and 2%, respectively, when the error size of the Best-Fit Extension problem is zero.

Therefore, for practical application, we suggest that *α*_1 _= 1% and *α*_2 _= 2% be used when the error size of the Best-Fit Extension problem is zero. We select the cutoff values such that the Boolean functions obtained by the CST-based BN are the same as those obtained by the original BN. However, we can use smaller cutoff values to reduce the number of false positive Boolean functions, because the original BN method tends to yield many false positive relationships, as shown in the simulation result.

In addition, a more careful dichotomization is required for a more accurate biological interpretation of the network structure. For example, since microarray data have continuous expression values with a considerably large amount of information, the dichotomization may require the selection of an appropriate threshold value depending on the biological function of each gene [35]. The performance may not be remarkable when a small number of time points and genes are available. However, we show that the proposed variable selection method is significantly more efficient for large-scale gene regulatory network studies. For example, the CST-based BN is approximately 114 times faster than the original BN for 800 genes (Figure 6).

The next step would be to perform a biological evaluation of the selected network structure. However, the main focus of our study is to improve the computation times of the BN by using the CST. Our approach allows the application of the BN to genome-wide network construction and discovery. A future study will evaluate the accuracy of the BN and compare it with other network methods such as the Bayesian network and hierarchical scale-free network.

## Methods

The proposed CST-based BN consists of two steps. The first step is to determine a pair of genes that are associated with each other. The second step is to determine the third gene that is conditionally associated with the pair of genes identified in the first step.

### First step for the main effect

Let *n *be the total number of genes. In the first step, 2 × 2 contingency tables are constructed from the dichotomized gene expression data. The *p*th row comprises the *i*th gene expression level at time *t *- 1 while the *q*th column of the table comprises the *j*th gene expression level at time *t *(*i *= 1, ..., *n*; *j *= 1, ..., *n*; *p *= 0,1; *q *= 0,1). For the *i*th and *j*th genes, a 2 × 2 contingency table is constructed with four cells: {0, 0}, {0,1}, {1, 0}, and {1,1}, where {*p*, *q*} represent the *i*th gene expression level at time *t *- 1 and the *j*th gene expression level at time *t*, respectively.

A CST statistic is then computed for testing the independence between two genes. For multinomial sampling with probabilities {*π*_*pq*_} in the contingency table, the null hypothesis of independence is *H*_0 _: *π*_*pq *_= *π*_*p*+_*π*_*q*+ _(the *i*th gene at time *t *- 1 and the *j*th gene at time *t *are independent) for all *p *(= 0,1) and *q *(= 0,1). The conventional Pearson's CST can be used to test *H*_0 _using the observed frequency *O*_*pq *_and the expected frequency *E*_*pq *_under *H*_0_. For the continuity correction, we add an arbitrary small number *a *to each observed frequency in order to prevent *E*_*pq *_from becoming zero [36]. We use *a *= 0.1 for the correction. Generally, {*π*_*p*+_} and {*π*_+*q*_} are unknown. The maximum likelihood (ML) estimates are the sample marginal proportions {π^
 MathType@MTEF@5@5@+=feaafiart1ev1aaatCvAUfKttLearuWrP9MDH5MBPbIqV92AaeXatLxBI9gBaebbnrfifHhDYfgasaacH8akY=wiFfYdH8Gipec8Eeeu0xXdbba9frFj0=OqFfea0dXdd9vqai=hGuQ8kuc9pgc9s8qqaq=dirpe0xb9q8qiLsFr0=vr0=vr0dc8meaabaqaciaacaGaaeqabaqabeGadaaakeaaiiGacuWFapaCgaqcaaaa@2E80@_*p*+ _= *O*_*p*+_/*O*_++_} and {π^
 MathType@MTEF@5@5@+=feaafiart1ev1aaatCvAUfKttLearuWrP9MDH5MBPbIqV92AaeXatLxBI9gBaebbnrfifHhDYfgasaacH8akY=wiFfYdH8Gipec8Eeeu0xXdbba9frFj0=OqFfea0dXdd9vqai=hGuQ8kuc9pgc9s8qqaq=dirpe0xb9q8qiLsFr0=vr0=vr0dc8meaabaqaciaacaGaaeqabaqabeGadaaakeaaiiGacuWFapaCgaqcaaaa@2E80@_+*q *_= *O*_+*q*_/*O*_++_}, where *O*_++ _= Σ_*p*_Σ_*q*_*O*_*pq*_. *E*_*pq *_is estimated as *E*_*pq *_= *O*_++_π^
 MathType@MTEF@5@5@+=feaafiart1ev1aaatCvAUfKttLearuWrP9MDH5MBPbIqV92AaeXatLxBI9gBaebbnrfifHhDYfgasaacH8akY=wiFfYdH8Gipec8Eeeu0xXdbba9frFj0=OqFfea0dXdd9vqai=hGuQ8kuc9pgc9s8qqaq=dirpe0xb9q8qiLsFr0=vr0=vr0dc8meaabaqaciaacaGaaeqabaqabeGadaaakeaaiiGacuWFapaCgaqcaaaa@2E80@_*p*+_π^
 MathType@MTEF@5@5@+=feaafiart1ev1aaatCvAUfKttLearuWrP9MDH5MBPbIqV92AaeXatLxBI9gBaebbnrfifHhDYfgasaacH8akY=wiFfYdH8Gipec8Eeeu0xXdbba9frFj0=OqFfea0dXdd9vqai=hGuQ8kuc9pgc9s8qqaq=dirpe0xb9q8qiLsFr0=vr0=vr0dc8meaabaqaciaacaGaaeqabaqabeGadaaakeaaiiGacuWFapaCgaqcaaaa@2E80@_+*q *_= *O*_*p*+_*O*_+*q*_/*O*_++_. Therefore, the chi-square statistic is expressed as follows:

χ2=∑p∑q(Opq−Epq)2Epq     (2)
 MathType@MTEF@5@5@+=feaafiart1ev1aaatCvAUfKttLearuWrP9MDH5MBPbIqV92AaeXatLxBI9gBaebbnrfifHhDYfgasaacH8akY=wiFfYdH8Gipec8Eeeu0xXdbba9frFj0=OqFfea0dXdd9vqai=hGuQ8kuc9pgc9s8qqaq=dirpe0xb9q8qiLsFr0=vr0=vr0dc8meaabaqaciaacaGaaeqabaqabeGadaaakeaaiiGacqWFhpWydaahaaWcbeqaaiabikdaYaaakiabg2da9maaqafabaWaaabuaeaadaWcaaqaaiabgIcaOiabd+eapnaaBaaaleaacqWGWbaCcqWGXbqCaeqaaOGaeyOeI0Iaemyrau0aaSbaaSqaaiabdchaWjabdghaXbqabaGccqGGPaqkdaahaaWcbeqaaiabikdaYaaaaOqaaiabdweafnaaBaaaleaacqWGWbaCcqWGXbqCaeqaaaaaaeaacqWGXbqCaeqaniabggHiLdaaleaacqWGWbaCaeqaniabggHiLdGccaWLjaGaaCzcamaabmaabaGaeGOmaidacaGLOaGaayzkaaaaaa@4BAE@

Using this CST, the significant genes are selected by an appropriate selection criterion *α*_1_. A further discussion on the appropriate choice of *α*_1 _is provided in the Result section.

### Second step for the conditional effect

Assume that the *i*th gene at time *t *- 1 is selected in the first step for the *j*th gene at time *t*. Then, a 2 × 2 × 2 contingency table can be constructed that consists of three genes – the *i*th and *j*th genes selected in the first step and an additional new gene *h *at time *t *- 1. This contingency table consists of eight cells: {0,0,0}, {0,0,1}, {0,1,0}, {0,1,1}, {1,0,0}, {1,0,1}, {1,1,0}, and {1,1,1}, where {*o*, *p*, *q*} represent the *h*th gene expression level at time *t *- 1, *i*th gene expression level at time *t *- 1, and *j*th gene expression level at time *t*, respectively (*i *= 1, ..., *n*; *j *= 1, ..., *n*; *h *= 1, ..., *n*; *o *= 0,1; *p *= 0,1; *q *= 0,1).

For the given expression value of *h*, there are two 2 × 2 contingency tables for the *i *and *j *genes. We focus on the conditional independence test. The null hypothesis that the *i*th gene at time *t *- 1 and the *j*th gene at time *t *are conditionally independent when the *h*th gene expression level at time *t *- 1 is given by *H*_0 _: *π*_*pq*|*o *_= *π*_*p*+|*o*_*π*_*q*+|*o *_for all *p *(= 0, 1) and *q *(= 0,1), where *π*_..|*o *_represents the conditional probability for the given *o*. We use the CST to test *H*_0 _using the observed frequency *O*_*opq *_and the expected frequency *E*_*opq *_under *H*_0_. We also add 0.1 to each observed frequency for the continuity correction. The ML estimates of *π*_*p*+|*o *_and *π*_+*q*|*o *_are the sample conditional proportions {π^
 MathType@MTEF@5@5@+=feaafiart1ev1aaatCvAUfKttLearuWrP9MDH5MBPbIqV92AaeXatLxBI9gBaebbnrfifHhDYfgasaacH8akY=wiFfYdH8Gipec8Eeeu0xXdbba9frFj0=OqFfea0dXdd9vqai=hGuQ8kuc9pgc9s8qqaq=dirpe0xb9q8qiLsFr0=vr0=vr0dc8meaabaqaciaacaGaaeqabaqabeGadaaakeaaiiGacuWFapaCgaqcaaaa@2E80@_*p*+|*o *_= *O*_*op*+_/*O*_*o*++_} and, {π^
 MathType@MTEF@5@5@+=feaafiart1ev1aaatCvAUfKttLearuWrP9MDH5MBPbIqV92AaeXatLxBI9gBaebbnrfifHhDYfgasaacH8akY=wiFfYdH8Gipec8Eeeu0xXdbba9frFj0=OqFfea0dXdd9vqai=hGuQ8kuc9pgc9s8qqaq=dirpe0xb9q8qiLsFr0=vr0=vr0dc8meaabaqaciaacaGaaeqabaqabeGadaaakeaaiiGacuWFapaCgaqcaaaa@2E80@_+*q*|*o *_= *O*_*o*+*q*_/*O*_*o*++_}, respectively where *O*_*o*++ _= Σ_*p*_Σ_*q*_*O*_*opq*_. *E*_*pq*|*o *_is estimated as *E*_*pq*|*o *_= *O*_*o*++_π^
 MathType@MTEF@5@5@+=feaafiart1ev1aaatCvAUfKttLearuWrP9MDH5MBPbIqV92AaeXatLxBI9gBaebbnrfifHhDYfgasaacH8akY=wiFfYdH8Gipec8Eeeu0xXdbba9frFj0=OqFfea0dXdd9vqai=hGuQ8kuc9pgc9s8qqaq=dirpe0xb9q8qiLsFr0=vr0=vr0dc8meaabaqaciaacaGaaeqabaqabeGadaaakeaaiiGacuWFapaCgaqcaaaa@2E80@_*p*+|*o*_π^
 MathType@MTEF@5@5@+=feaafiart1ev1aaatCvAUfKttLearuWrP9MDH5MBPbIqV92AaeXatLxBI9gBaebbnrfifHhDYfgasaacH8akY=wiFfYdH8Gipec8Eeeu0xXdbba9frFj0=OqFfea0dXdd9vqai=hGuQ8kuc9pgc9s8qqaq=dirpe0xb9q8qiLsFr0=vr0=vr0dc8meaabaqaciaacaGaaeqabaqabeGadaaakeaaiiGacuWFapaCgaqcaaaa@2E80@_+*q*|*o *_= *O*_*op*+_*O*_*o*+*q*_/*O*_*o*++_. Then, the chi-square statistic are given by

χo2=∑p∑q(Oopq−Eopq)2Eopq     (3)
 MathType@MTEF@5@5@+=feaafiart1ev1aaatCvAUfKttLearuWrP9MDH5MBPbIqV92AaeXatLxBI9gBaebbnrfifHhDYfgasaacH8akY=wiFfYdH8Gipec8Eeeu0xXdbba9frFj0=OqFfea0dXdd9vqai=hGuQ8kuc9pgc9s8qqaq=dirpe0xb9q8qiLsFr0=vr0=vr0dc8meaabaqaciaacaGaaeqabaqabeGadaaakeaaiiGacqWFhpWydaqhaaWcbaGaem4Ba8gabaGaeGOmaidaaOGaeyypa0ZaaabuaeaadaaeqbqaamaalaaabaGaeiikaGIaem4ta80aaSbaaSqaaiabd+gaVjabdchaWjabdghaXbqabaGccqGHsislcqWGfbqrdaWgaaWcbaGaem4Ba8MaemiCaaNaemyCaehabeaakiabcMcaPmaaCaaaleqabaGaeGOmaidaaaGcbaGaemyrau0aaSbaaSqaaiabd+gaVjabdchaWjabdghaXbqabaaaaaqaaiabdghaXbqab0GaeyyeIuoaaSqaaiabdchaWbqab0GaeyyeIuoakiaaxMaacaWLjaWaaeWaaeaacqaIZaWmaiaawIcacaGLPaaaaaa@5148@

for *o *= 0,1. We assume that the *i*th and *j*th genes are not independent from the first step. We select the *h*th gene if at least one of the two CST in the second step is significant (the p-value of the test is less than *α*_2_).

The rationale for using this conditional independence test is that *h *affects the association between two genes *i *and *j*. The conditional test approach is very effective in the determination of a relationship for more than two genes.

### Implementation of two step variable selection method

We select *n*_1, *j *_genes at time *t *- 1 that are associated with the *j*th gene at time *t *in the first step where 1 ≤ *n*_1, *j *_≤ *n*. If the *i*th gene is one of the *n*_1, *j *_genes, we select *n*_2, *ij *_genes in the second step for 1 ≤ *n*_2, *ij *_≤ *n *- 1 (excluding the *i*th gene). Then, we consider all possible combinations for selected three genes (when *k *= 3); one gene is the *i*th gene selected in the first step and the other two genes are selected in the second step. These combinations are used instead of all possible combinations in the original BN algorithms. The time complexity of determining the Boolean functions for the *j*th gene is *O*(22k⋅∑i=1n1,j⋅(n2,ijk)
 MathType@MTEF@5@5@+=feaafiart1ev1aaatCvAUfKttLearuWrP9MDH5MBPbIqV92AaeXatLxBI9gBaebbnrfifHhDYfgasaacH8akY=wiFfYdH8Gipec8Eeeu0xXdbba9frFj0=OqFfea0dXdd9vqai=hGuQ8kuc9pgc9s8qqaq=dirpe0xb9q8qiLsFr0=vr0=vr0dc8meaabaqaciaacaGaaeqabaqabeGadaaakeaacqaIYaGmdaahaaWcbeqaaiabikdaYmaaCaaameqabaGaem4AaSgaaaaakiabgwSixpaaqadabaGaeyyXIC9aaeWaaeaafaqabeGabaaabaGaemOBa42aaSbaaSqaaiabikdaYiabcYcaSiabdMgaPjabdQgaQbqabaaakeaacqWGRbWAaaaacaGLOaGaayzkaaaaleaacqWGPbqAcqGH9aqpcqaIXaqmaeaacqWGUbGBdaWgaaadbaGaeGymaeJaeiilaWIaemOAaOgabeaaa0GaeyyeIuoaaaa@4814@·*m*·*poly*(*k*)) and that of variable selection using the CST is *O*(*n*^2 ^+ *n*_1, *j*_·(*n *- 1)). Hence, the total time complexity of the proposed algorithm is expressed as follows:

O(n2+n1,j⋅(n−1))+O(22k⋅∑j=1n∑i=1n1,j⋅(n2,ijk)⋅m⋅poly(k))     (4)
 MathType@MTEF@5@5@+=feaafiart1ev1aaatCvAUfKttLearuWrP9MDH5MBPbIqV92AaeXatLxBI9gBaebbnrfifHhDYfgasaacH8akY=wiFfYdH8Gipec8Eeeu0xXdbba9frFj0=OqFfea0dXdd9vqai=hGuQ8kuc9pgc9s8qqaq=dirpe0xb9q8qiLsFr0=vr0=vr0dc8meaabaqaciaacaGaaeqabaqabeGadaaakeaacqWGpbWtcqGGOaakcqWGUbGBdaahaaWcbeqaaiabikdaYaaakiabgUcaRiabd6gaUnaaBaaaleaacqaIXaqmcqGGSaalcqWGQbGAaeqaaOGaeyyXICTaeiikaGIaemOBa4MaeyOeI0IaeGymaeJaeiykaKIaeiykaKIaey4kaSIaem4ta8KaeiikaGIaeGOmaiZaaWbaaSqabeaacqaIYaGmdaahaaadbeqaaiabdUgaRbaaaaGccqGHflY1daaeWbqaamaaqahabaGaeyyXIC9aaeWaaeaafaqabeGabaaabaGaemOBa42aaSbaaSqaaiabikdaYiabcYcaSiabdMgaPjabdQgaQbqabaaakeaacqWGRbWAaaaacaGLOaGaayzkaaaaleaacqWGPbqAcqGH9aqpcqaIXaqmaeaacqWGUbGBdaWgaaadbaGaeGymaeJaeiilaWIaemOAaOgabeaaa0GaeyyeIuoaaSqaaiabdQgaQjabg2da9iabigdaXaqaaiabd6gaUbqdcqGHris5aOGaeyyXICTaemyBa0MaeyyXICTaemiCaaNaem4Ba8MaemiBaWMaemyEaKNaeiikaGIaem4AaSMaeiykaKIaeiykaKIaaCzcaiaaxMaadaqadaqaaiabisda0aGaayjkaiaawMcaaaaa@77D9@

As *n *increases, the time complexity of determining the Boolean functions dominates the time complexity of variable selection because the former increases more rapidly than the latter.

### EXAMPLE

Table 3 shows the expression profiles of eight genes with 17 time points. Here, only two Boolean functions – *f*_1 _= ¬*G*3 ∨ (¬*G*4 ∧ ¬*G*8) and *f*_3 _= ¬*G*1 ∨ (*G*2 ∧ *G*6) – are true. To determine these functions, the original BN searches all possible combinations of genes at time *t *- 1 for every target gene at time *t *(_8_*C*_3 _× 8 = 448 when the indegree is three). We can reduce the BN combinations by using the proposed variable selection method. Table 4 shows the result obtained by using the proposed variable selection method. Five genes at time *t *(2nd, 4th, 5th, 6th, and 8th genes) are excluded because they do not have any genes selected in the first step.

**Table 3:** Example of dichotomized gene expression profiles

time	G1	G2	G3	G4	G5	G6	G7	G8
t1	1	0	0	0	1	0	1	1
t2	1	1	0	1	1	1	1	0
t3	1	1	1	1	1	1	0	1
t4	0	1	1	0	1	0	0	0
t5	1	1	1	0	0	0	1	1
t6	0	1	0	0	1	1	1	1
t7	1	0	1	1	0	1	1	1
t8	0	1	0	0	1	1	1	1
t9	1	1	1	0	1	0	1	1
t10	0	1	0	1	0	0	1	0
t11	1	0	1	0	1	0	0	1
t12	0	0	0	1	1	1	0	0
t13	1	0	1	1	0	1	0	0
t14	0	0	0	1	0	0	0	0
t15	1	0	1	0	1	0	0	1
t16	0	1	0	1	1	0	1	0
t17	1	0	1	0	0	1	0	1
t18	0	0	0	1	0	1	0	0

**Table 4:** Result of variable selection with 8 genes

First step			Second step			
*j*th gene(*t*)	*i*th gene(*t *- 1)	p-value	*h*th gene(*t *- 1)	p-value1	p-value2	combinations
G1	G1	0.0013	G5	0.0185	0.0252	_4_*C*_2 _= 6
			G6	0.0191	0.033	
			G7	0.0059	0.0656	
			G8	0.1049	0.0192	
	
	G3	0.0253	G2	0.0059	0.0208	_6_*C*_2 _= 15
			G4	0.0208	0.0059	
			G5	0.0185	0.0071	
			G6	0.0191	0.0058	
			G7	0.0414	0.0035	
			G8	0.1049	0.0021	

G3	G1	0.0013	G2	0.0065	0.0835	_6_*C*_2 _= 15
			G4	0.0035	0.1165	
			G5	0.0185	0.0252	
			G6	0.0033	0.1016	
			G7	0.033	0.0191	
			G8	0.1049	0.0192	
	
	G3	0.0078	G5	0.0185	0.1443	1

G7	G4	0.0078	G1	0.0102	0.2063	_5_*C*_2 _= 10
			G2	0.4774	0.0033	
			G3	0.0059	0.3535	
			G5	0.5469	0.0071	
			G8	0.0143	0.4864	

Total number of combinations						47

**Figure 7:**
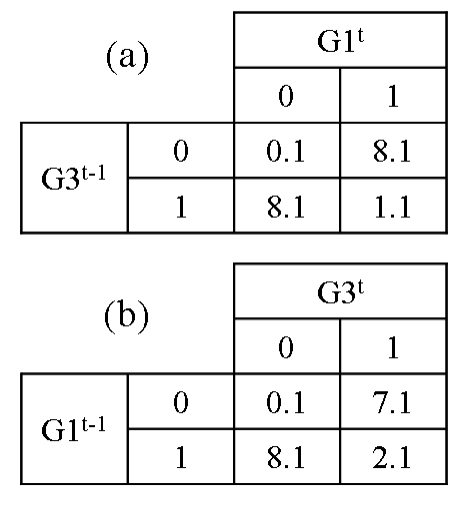
**First step for the main effect**. 2 × 2 contingency table with (a) *G*3 at time *t *and *G*1 at time *t *- 1 and (b) *G*3 and *G*1 at time *t *- 1. We added 0.1 to each cell for the continuity correction.

**Figure 8:**
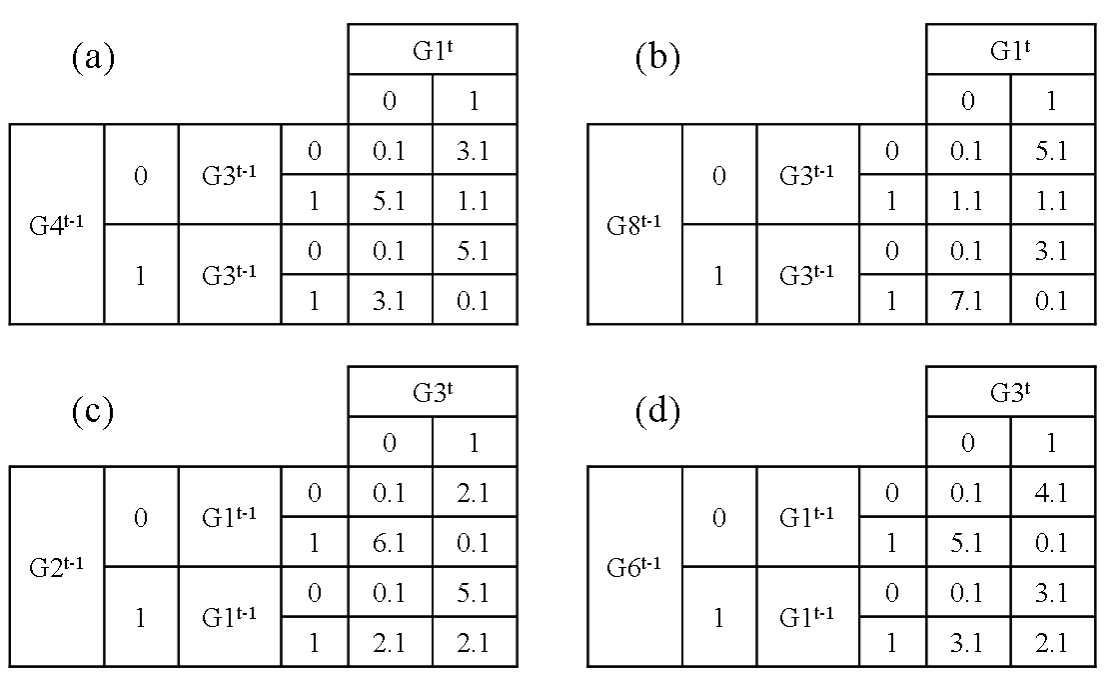
**Second step for the conditional effect**. 2 × 2 × 2 tables with three genes: a gene at time *t*, a gene at time *t *- 1 from the first step, and a new gene at time *t *- 1. (a) (*G*1^*t*^, *G*3^*t *- 1^, *G*4^*t *- 1^), (b) (*G*1^*t*^, *G*3^*t *- 1^, *G*8^*t *- 1^), (c) (*G*1^*t*^, *G*3^*t *- 1^, *G*2^*t *- 1^), and (d) (*G*1^*t*^, *G*3^*t *- 1^, *G*6^*t *- 1^).

Figures 7(a) and 7(b) show the 2 × 2 contingency tables using (*G*1^*t*^, *G*3^*t *- 1^) and (*G*3^*t*^, *G*1^*t *- 1^), respectively. The second step is performed only for the selected genes in the first step in order to identify genes that are conditionally associated with the target gene. Figure 8 shows 2 × 2 × 2 tables with three genes – a gene at time *t*, a gene at time *t *- 1 from the first step, and new gene at time *t *- 1: (a) for (*G*1^*t*^, *G*3^*t *- 1^, *G*4^*t *- 1^), (b) for (*G*1^*t*^, *G*3^*t *- 1^, *G*8^*t *- 1^), (c) for (*G*1^*t*^, *G*3^*t *- 1^, *G*2^*t *- 1^), and (d) for (*G*1^*t*^, *G*3^*t *- 1^, *G*6^*t *- 1^). The total number of possible combinations of the CST-based BN is 47. Therefore, the total time complexity of the CST-based BN is 9.5 times faster than that of the original BN.

## Availability and requirements

Project name: Boolean networks for Large-scale gene regulatory network Project home page: 

## Supplementary Material

Additional file 1 Boolean functions for a toy example. R code for true Boolean functions in a toy example. Click here for file

Additional file 2 Data set for toy example. Binary data set of 18 time points × 8 genes generated by using the two Boolean functions. Click here for file

Additional file 3 27 Boolean functions for the simulation study. R code for true functions in the simulation study. Click here for file

Additional file 4 Data set for the simulation study. 43 data set of 7 time points × 40 genes generated by using 27 Boolean functions Click here for file

Additional file 5 Yeast cell cycle data. Binary data set of randomly selected 40–120 genes. The Each data set consist of time points (row) number of genes (column). The mean value was used as a dichotomization criterion. Click here for file
